# Circulating angiogenic stem cells in type 2 diabetes are associated with glycemic control and endothelial dysfunction

**DOI:** 10.1371/journal.pone.0205851

**Published:** 2018-10-15

**Authors:** Nagma Zafar, Sathya S. Krishnasamy, Jasmit Shah, Shesh N. Rai, Daniel W. Riggs, Aruni Bhatnagar, Timothy E. O’Toole

**Affiliations:** 1 Department of Medicine, Diabetes and Obesity Center, University of Louisville, Louisville, Kentucky, United States of America; 2 Department of Medicine, Division of General Pediatrics, University of Louisville, Louisville, Kentucky, United States of America; 3 Department of Medicine, Division of Endocrinology, Metabolism and Diabetes University of Louisville, Louisville, Kentucky, United States of America; 4 Department of Internal Medicine, Aga Khan University, Nairobi, Kenya; 5 Department of Bioinformatics and Biostatistics, University of Louisville, Louisville, Kentucky, United States of America; 6 Envirome Institute, University of Louisville, Louisville, Kentucky, United States of America; University of Kansas Medical Center, UNITED STATES

## Abstract

Circulating angiogenic cells (CACs) of various described phenotypes participate in the regeneration of the damaged endothelium, but the abundance of these cells is highly influenced by external cues including diabetes. It is not entirely clear which CAC populations are most reflective of endothelial function nor which are impacted by diabetes. To answer these questions, we enrolled a human cohort with variable CVD risk and determined relationships between stratified levels of CACs and indices of diabetes and vascular function. We also determined associations between CAC functional markers and diabetes and identified pro-angiogenic molecules which are impacted by diabetes. We found that subjects with low levels of CD34^+^/AC133^+^/CD31^+^/CD45^dim^ cells (CAC-3) had a significantly higher incidence of diabetes (p = 0.004), higher HbA1c levels (p = 0.049) and higher CVD risk scores. Furthermore, there was an association between low CAC-3 levels and impaired vascular function (p = 0.023). These cells from diabetics had reduced levels of CXCR4 and VEGFR2, while diabetics had higher levels of certain cytokines and pro-angiogenic molecules. These results suggest that quantitative and functional defects of CD34^+^/AC133^+^/CD31^+^/CD45^dim^ cells are associated with diabetes and vascular impairment and that this cell type may be a prognostic indicator of CVD and vascular dysfunction.

## Introduction

Type 2 diabetes has become a world-wide epidemic affecting all populations regardless of race, gender and socioeconomic status. While the pathophysiological manifestations of diabetes are multiple and diverse, cardiovascular tissues appear to be particularly vulnerable to hyperglycemia. Indeed diabetes is strongly associated with vascular dysfunction [1–3] and impaired angiogenesis [4, 5], and is a risk factor for cardiovascular disease (CVD) and adverse event [6, 7]. Despite these well-recognized associations, the mechanisms whereby diabetes impairs angiogenesis and vascular function are not completely understood.

In healthy individuals, post-natal angiogenesis is facilitated by endothelial progenitor cells (EPCs), initially described by Asahara et. al. in 1997[8]. These peripheral blood cells expressed both a stem cell antigen (CD34) and an endothelial antigen (Flk-1:VEGFR2:KDR). When cultured *in vitro*, these cells acquired additional endothelial antigen expression and tube formation capability. *In vivo*, injected EPCs were found to incorporate into vascular structures. Since this initial description, other vascular reparative cells of varying phenotypes have been isolated from cellular fractions of peripheral blood, cord blood and bone marrow [9]. In general, these cells are mobilized by tissue injury or hypoxic signals, home to the site of injury and participate in its repair by terminal differentiation or paracrine effects [9]. The salubrious effects of these circulating angiogenic cells (CACs) have been well documented and their efficacy in promoting vascular perfusion and tissue repair has been demonstrated in multiple animal studies [10–15]. Supporting this role in promoting a functional endothelium, low CAC levels are predictive of cardiovascular events and mortality in subjects with coronary artery disease and CAC levels have been shown to have a negative correlation with early endothelial dysfunction as measured by flow mediated dilation of the brachial artery [16, 17].

However, the abundance and functionality of CACs is impacted by multiple external factors including concurrent disease, environmental exposures, and lifestyle features such as diet and level of exercise [18–23]. Furthermore, diabetes, a major risk factor for CVD, has been associated in several studies with quantitative and qualitative defects in CACs [24–30]. However, quantification in these studies described diverse cell types as determined by flow cytometry using a varied and often limited set of markers. Other studies of CACs in diabetes quantified these cells by culture methods where colony forming units may have derived from initial monocytes that only acquired endothelial antigen expression from *ex vivo* culture conditions [9]. These limitations are important, because only by knowing which specific subpopulations are most affected and which are associated with vascular dysfunction, can it be possible to better understand the mechanisms underlying this deficit and identify the locus of CAC production and development that might be affected by diabetes. Such measurements may also identify high risk individuals and high risk populations that might be more susceptible to diabetes-induced vascular dysfunction.

Therefore, we examined, for the first time, a full array of CACs in a cohort of subjects with variable CVD risk, and examined their relationships to vascular function, diabetes, and demographic features. We found that subjects with lower levels of CD34^+^/AC133^+^/CD31^+^/CD45^dim^ cells tended to have a higher prevalence of diabetes, higher HbA1c levels and higher CVD risk scores. Some ethnic differences were identified. Furthermore, these cells in diabetics are predicted to be less responsive to mobilizing cues as they demonstrated a reduction of CXCR4 expression. Conversely, those subjects with high levels of these cells had better endothelial function as measured by peripheral arterial tonometry. Thus, the depletion of CD34^+^/AC133^+^/CD31^+^/CD45^dim^ cells is predictive of endothelial dysfunction. Levels of these cells can be used as a specific indicator of declining vascular health and this knowledge allows a greater window of opportunity for initiation of preventive care measures that retard progression to the disease state.

## Materials and methods

### Study cohort and clinical procedures

Subjects between the ages of 22 and 65 years were recruited from the Diabetes and Primary Care Clinics of the University of Louisville. The study period extended from June 2011 to May 2013. All subjects signed informed consents and the Institutional Review Board of the University of Louisville approved the study. Subjects were prescreened, and those with conditions known to effect peripheral blood cell counts and bone marrow function were excluded from the study. These conditions included history of malignancies, organ transplant, renal replacement therapy, type 1 diabetes, untreated thyroid disease, anemia, acute infections, HIV infection, hepatitis, and unhealed wounds. Subjects on hormone replacement therapy or medications affecting bone marrow function or peripheral blood cell counts were also excluded from the study. Other excluded subjects included those unwilling or unable to provide informed consent, pregnant or lactating women, prisoners and other vulnerable populations. A total of 108 subjects participated in the study; of these, 3 were not used in the final analysis due to technical errors in sample processing.

Enrolled patients fasted for a minimum of eight hours. All menstruating females were in the luteal phase of their cycle. At the clinic, subjects completed a health and diabetes questionnaire, biometrics were recorded, and urine and blood samples were collected. Peripheral endothelial function (reactive hyperemia index: RHI) was measured using the EndoPAT, according to the guidelines recommended by the manufacturer (Itamar Medical). In brief, after baseline recordings of the pulse wave amplitude from a fingertip on each arm, supra systolic pressure was applied to the non-dominant upper arm by a blood pressure cuff. The cuff was deflated after 5 minutes and reactive hyperemia recorded by the change in finger arterial pulse volume amplitude (PVA). The ratio between hyperemic and baseline PVA was normalized to the same ratio in the contralateral arm and reported as the reactive hyperemia index (RHI).

### Measures of obesity

Three methods were used to account for body size and fat distribution: body mass index (BMI), waist to hip ratio (WHR) and percent body fat. BMI was calculated using the formula: weight (lb) / [height (in)]^2^ x 703, with BMI of ≥ 30 reported as obese. Waist and hip measurements were performed in accordance with the WHO 2008 Expert Consultation Report [31]. The cut off for high WHR was taken from the recommendations in the same report, where abdominal obesity is defined as a WHR above 0.90 for males and 0.85 for females. Body fat percentage was measured using a hand held bioelectrical impedance instrument from Omron, model BF306 and expressed as a percent of the body weight.

### CVD risk score calculations

Ten year atherosclerotic cardiovascular disease (ASCVD) risk scores of heart disease or stroke were calculated using the algorithm published in the 2013 ACC/AHA Guideline on the Assessment of Cardiovascular Risk [32]. The sum of CVD risk factors was calculated by assigning a score of +1 for each of the following features: age > 40 years, male gender, diagnosis of hypertension or on medications for hypertension, LDL cholesterol of >130 mg/dl, current smoker, and diabetes.

### CAC analysis

Eight ml of venous blood collected in a CPT Mononuclear Cell preparation tube (Becton Dickinson) was processed within 3 hours of collection. Mononuclear cells were separated by centrifugation at 1700*g* for 30 minutes. The buffy coat layer was collected and washed twice with PBS+1%BSA. Cells were pelleted by centrifugation at 400*g* for 5min and incubated with Fc Block for 10 minutes (Miltenyi Biotec). 50ul aliquots were then incubated with FITC anti-CD41a (BD Biosciences), FITC-CD235a (eBioscience), FITC-CD16 (eBioscience), QDot655anti-CD14 (Invitrogen), PE-CF594 anti-CD34 (BD Biosciences), APC anti- AC133 (Miltenyi Biotec), V450 anti-CD31 (BD Biosciences), V500 anti-CD45 (BD Biosciences), PECy7-CXCR4 (eBioscience) and Alexa Fluor 700 anti-VEGFR2 (R&D Systems). In addition, we included a discriminator of cell viability (Live Dead Green; Becton Dickinson). An unstained cell aliquot was used as a control. After a 30 min incubation, the cells were washed, resuspended in PBS+1%BSA, and 50ul of volumetric counting beads (Accucount Particles; Spherotech) added. The samples then run on an LSR II flow cytometer (Becton Dickinson) set to acquire 20,000 beads. Collected events were analyzed using FloJo software. Initial gating selected a lymphocyte population that was negative for CD235a, CD41a, CD16, CD14 and the viability marker. Eighteen distinct CAC subgroups were then identified by the variable expression of CD31, CD45, CD34, AC133, and KDR (S1 Table). Positive boundaries for gating were accomplished with the unstained sample. CAC numbers were quantified and normalized to volume of blood using bead counts. The median fluorescence intensity of positive staining for CXCR4, VEGFR2 and IR in the CAC-3 subgroup was calculated using FacsDiva software and expressed in arbitrary units.

### Measurement of whole blood and plasma analytes

Plasma was separated from whole blood anti-coagulated with EDTA by centrifugation at 1500g for 10 minutes and stored at -80°C until further use. Plasma glucose, insulin, a lipid profile (total cholesterol, HDL, LDL), and creatinine were measured using the Cobas Mira Chemistry System (Roche Diagnostic Systems, Inc.). Glucose was measured by the hexokinase assay (Sekisui Diagnostics, Charlottetown, Canada), insulin by the immunoturbidimetric assay (Kamiya Biomedical Company, Seattle, WA), LDL-C, HDL-C and triglycerides were measured by enzyme assays using calibrators from Wako Diagnostics (Richmond, VA). Total cholesterol was measured similarly with calibrators and controls from Thermo Electron. Creatinine was measured by the Jaffe reaction using alkaline picarate obtained from Thermo Electron. hsCRP was measured using the VITROS Chemistry Products at a CLIA certified lab. HbA1c was measured using the Bayer A1c Now kit. Complete blood cell counts were determined with the Abbott Cell Dyne.

### Statistical analysis

Descriptive statistics are presented as mean ± SD for continuous variables and as frequencies and percentages for categorical variables. Subjects were divided into high and low CAC count groups using the median value. Data was analyzed using a Chi-square test, a two-sample rank sum test, and univariable and multivariable logistic regressions using SAS software (Version 9.4, SAS Institute, Inc., Cary, North Carolina). Linear regressions and the predicted value of mean of the CAC count from the complete model were obtained using SPSS software (Version 21, Chicago, IL, USA). Values of *P* < 0.05 were considered statistically significant.

## Results

### CAC levels and cohort characteristics

While several populations of CACs have been described, it is unclear which are most predictive of diabetes, which are most reflective of endothelial function, and if the expression of additional phenotypic markers are altered in diabetics. To begin to gain insight into these questions, we examined relationships between CAC levels and diabetes in a cohort of 105 subjects stratified into high and low cell count groups based on median values. We observed that subjects with diabetes were more prominent in the low count group of 6 CAC subpopulations (CAC-5, CAC-6, CAC-9, CAC-11, CAC-14, and CAC-17), which were significantly associated with diabetes at p<0.05 (Table 1). Next, to determine which CAC subpopulations were most reflective of endothelial function, we stratified high and low count groups versus RHI values. Only CAC-3 (CD34^+^/AC133^+^/CD31^+^/CD45^dim^) demonstrated a statistically significant positive association with RHI (Table 2). All further analysis was done with this CAC subpopulation as it was most reflective of endothelial health.

**Table 1:** Circulating angiogenic cells (CACs) and diabetes prevalence.

**CAC**	**Diabetics n (%) in High Count Group n = 52**	**Diabetics n (%) in Low Count Group n = 53**	**P Value**
CAC-1	20 (39)	27 (51)	0.198
CAC-2	24 (46)	23 (43)	0.776
CAC-3	19 (37)	28 (53)	0.093
CAC-4	21 (40)	26 (49)	0.372
CAC-5	16 (31)	31 (59)	0.004*
CAC-6	17 (33)	30 (57)	0.014*
CAC-7	21 (40)	26 (49)	0.372
CAC-8	25 (48)	22 (42)	0.499
CAC-9	18 (35)	29 (55)	0.038*
CAC-10	20 (39)	27 (51)	0.198
CAC-11	17 (33)	30 (57)	0.014*
CAC-12	20 (39)	27 (51)	0.198
CAC-13	21 (40)	26 (49)	0.372
CAC-14	18 (35)	29 (55)	0.038*
CAC-15	21 (40)	26 (49)	0.372
**KDR^+^ Phenotypes**	**Diabetics n (%) in High Count Group n = 44**	**Diabetics n (%) In Low Count Group n = 43**	**P value**
CAC-16	15 (34)	20 (47)	0.238
CAC-17	13 (30)	22 (51)	0.040*
CAC-18	18 (41)	17 (40)	0.896

* denotes p < 0.05

**Table 2:** CACs and reactive hyperemia index (RHI).

CAC*	RHI (n = 100)	RHI in High Count Group	RHI in Low Count Group	P Value
CAC-3	2.1 ± 0.6	2.25 ± 0.6	2.02 ± 0.7	0.03**
CAC-5	2.1 ± 0.6	2.25 ± 0.7	2.03 ± 0.6	0.10
CAC-6	2.1 ± 0.6	2.25 ± 0.7	2.03 ± 0.6	0.10
CAC-9	2.1 ± 0.6	2.21 ± 0.6	2.07 ± 0.6	0.23
CAC-11	2.1 ± 0.6	2.20 ± 0.7	2.07 ± 0.6	0.30
CAC-14	2.1 ± 0.6	2.19 ± 0.6	2.08 ± 0.7	0.29
CAC-17	2.1 ± 0.7	2.16 ± 0.7	2.08 ± 0.6	0.73

*CACs from Table 1 associated with diabetes (p<0.1)

p values are calculated based on the Mann-Whitney U Test.

** p < 0.05

We performed a further demographic and biochemical analysis of the study cohort after stratification into those participants with high levels (52 subjects) and low levels (53 subjects) of CAC-3 (Table 3). The mean, median and range of CAC-3 counts per ml of blood for the entire cohort was 86, 46, and 0–919, respectively. The mean ± SD of the low cell group was 19 ± 13, while that of the high cell group was 154 ± 14 per ml blood. The two groups had similar characteristics except that subjects in the high count group had a greater mean percent body fat, although BMI and waist to hip ratio were not different in the two groups. The groups also differed in ethnicity, wherein African-Americans were predominantly in the high cell count group. Subjects with diabetes tended to be in the low cell count group, and in the unadjusted association, this was at a significance level of less than 10%.

**Table 3:** Demographics and characteristics of the study cohort stratified by CAC-3 level.

	Total (n = 105)	High Count (n = 52)	Low Count (n = 53)	*P* Value
Age (years)	48 ± 11	48 ± 11	48 ± 11	0.77
Female	61 (58)	35 (67)	26 (49)	0.06
Ethnicity				0.02*
Caucasian	62 (59)	28 (54)	34 (64)	0.28
African American	31 (30)	21 (40)	10 (19)	0.02*
Asian	4 (4)	0 (0)	4 (8)	
Latino	3 (3)	2 (4)	1 (2)	
Others	5 (5)	1 (2)	4 (8)	
Diabetes	47 (45)	19 (37)	28 (53)	0.09
Duration of Diabetes (years)	10 ± 8	11 ± 8	9 ± 9	0.44
Hyperlipidemia	41 (39)	21 (40)	20 (38)	0.78
Hypertension	55 (52)	26 (50)	29 (55)	0.63
BMI ≥ 30	62 (59)	34 (65)	28 (53)	0.19
High WHR	76 (72)	38 (73)	38 (72)	0.75
Body Fat Percentage †	34 ± 9	37 ± 8	32 ± 10	0.02*
hsCRP ≥ 3 mg/L ‡	30 (29)	20 (39)	10 (19)	0.15
Current smoker	21 (20)	14 (27)	7 (13)	0.08
10 year ASCVD Risk Score §	7.7 ± 10.2	8.7 ± 12.0	6.5 ± 7.3	0.68
Sum of CVD Risk Factors ‡	2.7 ± 1.5	2.6 ± 1.4	2.9 ± 1.6	0.31
HbA1c %	6.87 ± 2.1	6.71 ± 2.0	7.03 ± 2.2	0.76
Fasting Plasma Glucose mg/dl ‡	140 ± 74	136 ± 69	144 ± 80	0.79
Fasting Plasma Insulin μIU/ml ‡	24 ± 20	24 ± 19	23 ± 22	0.36
HOMA-IR Score ‡	3.40 ± 3.5	3.39 ± 2.6	3.42 ± 4.3	0.52
Total Cholesterol mg/dl ‡	191 ± 46	190 ± 45	192 ± 49	0.94
LDL-Cholesterol mg/dl ‡	103 ± 31	101 ± 28	105 ± 35	0.55
HDL-Cholesterol mg/dl ‡	48 ± 16	49 ± 19	47 ± 11	0.73
Triglycerides mg/dl ‡	145 ± 129	142 ± 128	149 ± 133	0.76
White blood cell count K/μL ‖	5.5 ± 1.9	5.9 ± 2.0	5.2 ± 1.7	0.09
Lymphocyte count K/μL ‖	1.7 ± 0.6	1.8 ± 0.6	1.6 ± 0.6	0.11
Plasma Creatinine mg/dl ‡	0.98 ± 0.2	0.99 ± 0.2	0.96 ± 0.2	0.74
Endothelial Function †				
Reactive Hyperemia Index	2.1± 0.6	2.3 ± 0.6	2.0 ± 0.7	0.03*
`Medications				
Insulin	31 (30)	14 (27)	17 (32)	0.56
Metformin	37 (35)	16 (31)	21 (40)	0.34
Statins	37 (35)	19 (37)	18 (34)	0.78
β Blockers	23 (22)	13 (25)	10 (19)	0.49
ACE Inhibitors	32 (31)	19 (37)	13 (25)	0.18
Diuretics	37 (35)	16 (31)	21 (40)	0.34
Aspirin	33 (31)	12 (23)	21 (40)	0.07

Data are presented as mean ± SD and as n (% in column). Abbreviations: BMI—body mass index in kg/m^2^, hsCRP—highly sensitive C reactive protein, ASCVD—atherosclerotic cardiovascular disease, HOMA-IR–homeostasis model assessment -insulin resistance.

* *P* Value *<* 0.05,

^†^ n = 100 subjects,

^‡^ n = 93 subjects,

^§^ n = 86,

^‖^ n = 98.

We refined our analysis in a logistic regression model. As percent body fat and African American ethnicity had significant associations with CAC-3 levels in the univariable analysis (Table 3), these variables were included for adjustment in the multivariable logistic regression model to clarify the association between diabetes and cell counts. The results of this analysis indicate that subjects with diabetes were 4.4 times more likely to have a low cell count than those who did not have diabetes (Table 4). This association was present regardless of race, as in the model, the interaction of race and diabetes is only marginally significant (p = 0.07), which may be due to small sample size within each subgroup. To further investigate the association between race and CAC counts, we stratified the study population by race and analyzed the relationship between cell count and diabetes (S1 Table). This analysis showed that 90% of the African Americans in low cell count group had diabetes, while only 38% of African Americans in the high cell count group had diabetes (p = 0.007). Such disparities were not found in the Caucasian population, wherein 50% of Caucasians in the low cell count group had diabetes and 36% of Caucasians in the high cell count group had diabetes (p = 0.26), suggesting the influence of race and diabetes on CAC counts.

**Table 4:** Logistic regression models of the low CAC-3 count group and outcome variables.

Outcome	*P* value	Regression Coefficient	Odds Ratio	95% CI
Diabetes	0.004*	1.483	4.41	1.609–12.071
RHI	0.023*	1.042	2.84	1.156–6.952
HbA1c	0.049*	1.039	2.83	1.005–7.953
Glucose	0.074	0.006	1.01	0.999–1.013
HOMA-IR	0.421	0.055	1.06	0.925–1.206
Insulin	0.739	0.004	1.01	0.981–1.027
Sum of CVD risk factors	0.222	0.201	1.22	0.886–1.687
ASCVD score	0.943	0.002	1.00	0.940–1.069

*: p<0.05

All models were adjusted for ethnicity (African American) and body fat percentage.

### CAC-3 level, glycemic indices and, cardiovascular disease risk

**Fig 1:**
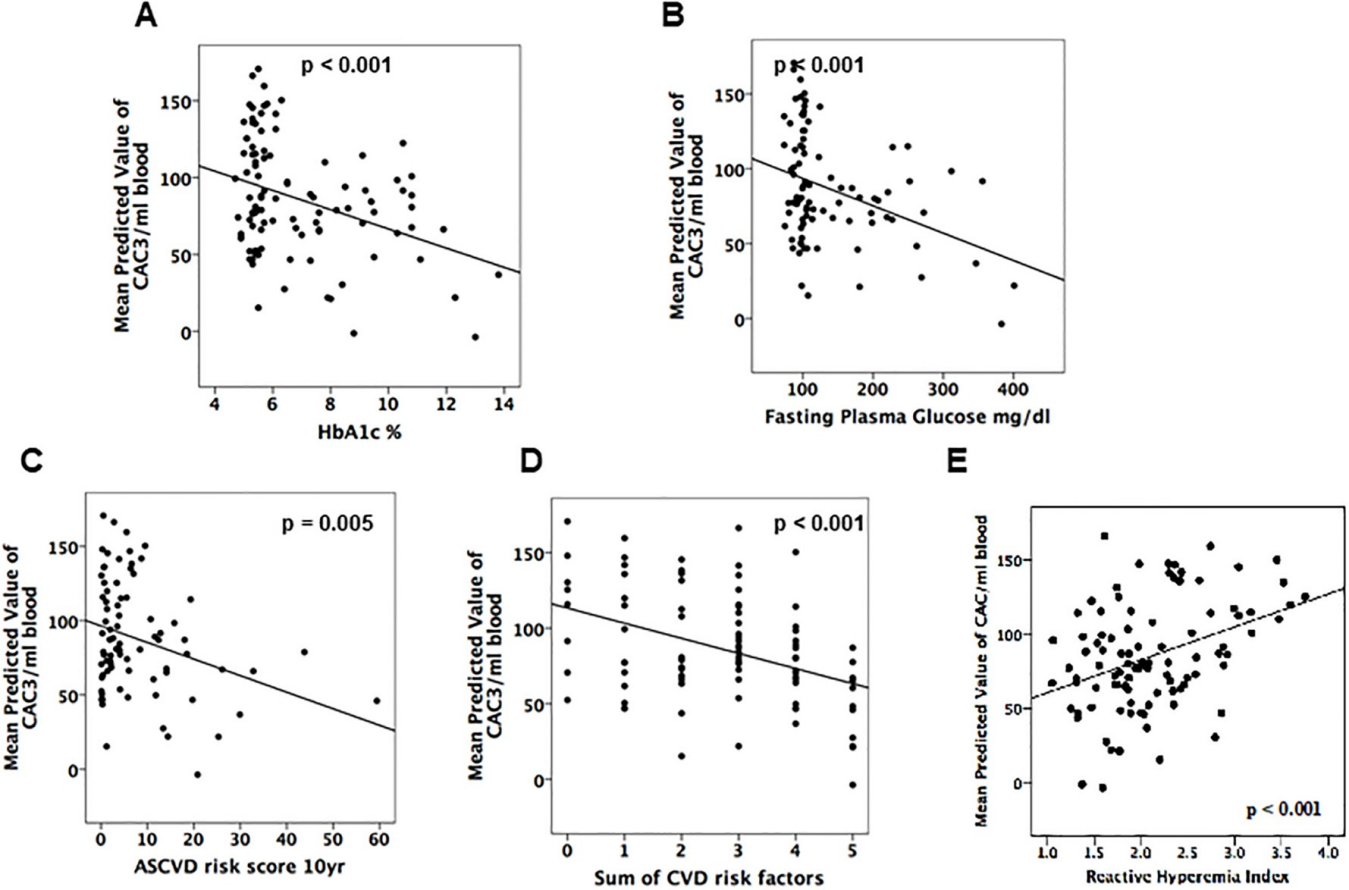
CAC-3 levels, glycemic indices and CVD risk. In continuous regression analyses, adjusted CAC-3 counts show significant negative correlations with HbA1c (A), fasting plasma glucose (B), ASCVD score (C) and sum of CVD risk factors (D). In a continuous regression analysis between adjusted CAC-3 counts and RHI value, a significant positive correlation was observed (E).

To further define the relationship between CAC-3 levels and diabetes, we determined associations between the low CAC-3 count group and HbA1c (> 5.6%), fasting plasma glucose, HOMA-IR, and insulin (Table 4). Of these markers of diabetes, a significant association with the low cell count group was only found in subjects with high HbA1c. These subjects were 2.8 times more likely to belong to the low CAC cell count group. Fasting glucose levels, insulin sensitivity (HOMA-IR) and insulin levels did not significantly impact CAC counts. The linear associations of glycemic indices were also examined with the adjusted CAC-3 counts. In the continuous regression analysis both HbA1c (Fig 1A) and fasting plasma glucose (Fig 1B) show significant negative correlations, indicating high glucose levels are associated with low CAC-3 counts. Neither insulin levels nor insulin sensitivity (HOMA-IR) were associated with CAC-3 counts.

We next examined the utility of CAC-3 levels in predicting CVD risk. We found that the widely used scores predictive of cardiovascular disease (the ASCVD risk score and sum of CVD risk factors) showed negative correlations with adjusted CAC-3 counts (Fig 1C and 1D). We also examined associations with the reactive hyperemia index (RHI), an indicator of endothelial function where higher scores indicate a healthier and more functional endothelium and lower scores are associated with CVD risk factors. We found that RHI scores were greater in the high cell count group (Tables 2 and 3). This association was also manifest in the regression of adjusted CAC-3 counts and RHI, confirming the positive correlation between CAC-3 counts and endothelial function (Fig 1E). Thus CAC-3 levels are inversely correlated with hyperglycemia and are predictive of CVD risk.

### CAC-3 functional assessment

**Fig 2:**
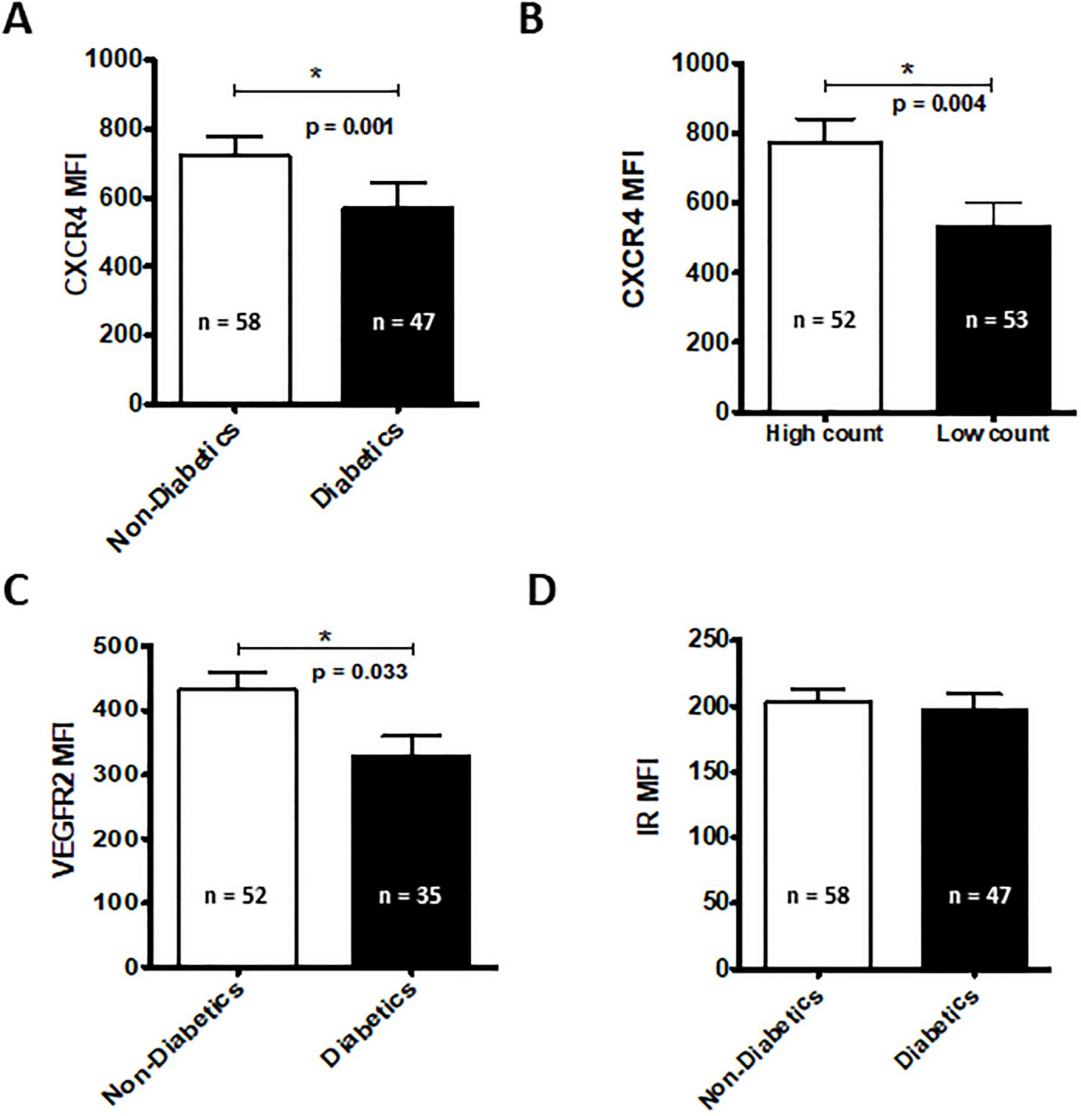
CAC-3 surface molecule expression. Levels of CXCR4 (A, B) VEGFR2 (C) and the insulin receptor (D) were quantified on CAC-3 cells by flow cytometry. n = 35–58.

In addition to quantitative correlations between diabetes and CAC-3 levels, hyperglycemia may also affect aspects of CAC-3 function. SDF-1α is a chemokine which, through interactions with its receptor CXCR4, enables the mobilization of CACs from the bone marrow and their targeted migration to distal tissues. Thus, CXCR4 expression is indicative of responsiveness to mobilizing cues. When we examined the expression of CXCR4 on CAC-3 cells, we found decreased expression in cells derived from diabetics (Fig 2A). CXCR4 expression levels were likewise decreased in the low cell count group (Fig 2B). CACs are also mobilized in response to VEGF. Thus, to further assess the responsiveness of CAC-3 cells, we quantified the levels of the VEGF receptor, VEGFR2. We found that levels of this receptor were lower in diabetics compared to non-diabetics (Fig 2C). No changes were observed in the expression of the insulin receptor in either study group (Fig 2D).

### Pro-angiogenic molecules and diabetes

In addition to quantitative and qualitative CAC defects, impaired angiogenesis in diabetics may result from decreased expression of pro-angiogenic cytokines or molecules. Thus we measured the levels of several plasma analytes in our cohort and determined association of stratified levels with diabetes. We observed that levels of angiopoietin-1, angiopoietin-2, VEGF-A, PIGF, EPO, HGF, sICAM-1, sE-selectin, and MMP-9 were higher in the diabetic group than in the non-diabetic group (Table 5). There was no statistically significant difference in stratified levels of SDF-1α with diabetes. Thus impairments in CACs, and CAC-3 in particular, rather than deficits in pro-angiogenic molecule expression may underlie impaired angiogenesis in diabetics.

**Table 5:** Stratified plasma analytes and diabetes.

Analyte	High analyte level Diabetics n (%)	Low analyte level Diabetics n (%)	P value
Angiopoietin-1	20 (50.0)	10 (25.0)	0.021
Angiopoietin-2	21 (52.5)	9 (22.5)	0.006
VEGF-A	24 (60.0)	6 (15.0)	<0.001
PIGF	21 (51.2)	9 (22.5)	0.009
EPO	23 (47.9)	7 (21.9)	0.018
HGF	26 (65.0)	4 (10.0)	<0.001
sICAM-1	21 (52.5)	9 (22.5)	0.006
sE-selectin	22 (55.0)	8 (20.0)	0.001
MMP-9	19 (47.5)	11 (27.5)	0.065
SDF-1α	16 (44.4)	20 (55.6)	0.302

## Discussion

**Fig 3:**
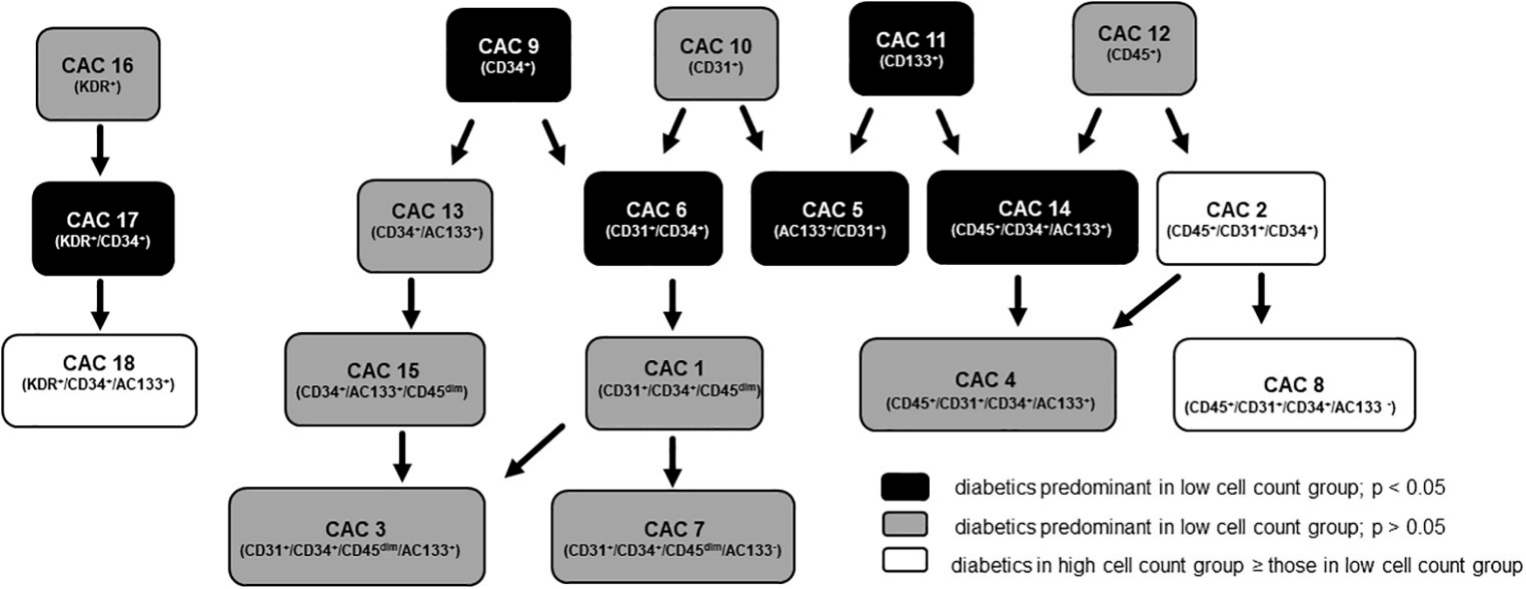
Summary of CACs and their association with diabetics. Illustrated is a schematic hierarchy of CAC subgroups and the abundance of diabetics in stratified low cell count groups.

In this study, using a cohort of individuals with CVD or at risk of developing CVD, we found that low levels of several CAC phenotypes were predictive of diabetes (Fig 3). However, only low levels of the CAC-3 subtype (CD34^+^/AC133^+^/CD31^+^/CD45^dim^) were associated with a higher incidence of diabetes, higher HbA1c levels, higher CVD risk scores and measured impairments in vascular function. Furthermore, by virtue of decreased CXCR4 and VEGFR2 expression, CAC-3 cells may be less responsive to mobilizing or functional cues from SDF-1α and VEGF. These results suggest that quantitative assessment of this cell type may be predictive of endothelial function and CVD. Using quantitative estimates of these cells as an early indicator of vascular dysfunction will allow a greater window of opportunity for initiation of preventive care measures that retard progression to the disease state.

Several prior animal [25, 28, 33] and human [24, 26, 27, 29] studies have demonstrated an inverse relationship between CAC levels and incidence of diabetes. However, as these studies pre-selected the diabetic phenotype and used variable markers to identify CACs, it remains unclear which cell phenotype is most predictive of diabetes. In addition, it is not even clear which CAC type might be best reflective of endothelial function. Using the at risk cohort described in this study, we found that low levels of several CAC subgroups (CAC-3, CAC-5, CAC-6, CAC-9, CAC-11, CAC-14, CAC-17), were associated with an increased incidence of diabetes. However only one cell type predictive of diabetes, CAC-3 (CD34^+^/AC133^+^/CD31^+^/CD45^dim^), was also positively associated with RHI score; thus high levels of cells were associated with a high RHI score and a healthy endothelium. It is interesting that an analogous cell type only lacking AC133 expression (CAC7: CD34^+^/AC133^-^/CD31^+^/CD45^dim^) displayed associations with neither diabetes outcomes or endothelial function. Thus “early” stem cells expressing the AC133 antigen seem essential for promoting vascular perfusion and endothelial function. Consistent with this idea, AC133^+^ cells have been shown to enhance re‐endothelialization of vascular lesions when transplanted into mice [34] and levels of these cells are inversely correlated with CVD risk and severity [17]. Hence, a reduced level of these cells in diabetes is indicative of poor vascular repair capacity and their measurement may be useful as a clinical marker of disease progression. Similarly, acid sphingomyelinase (ASM) activation in CD34^+^ reparative cells of diabetics is associated with their impaired functionality [29] and measurements of ASM activity can likewise be used to predict vascular repair capacity and vascular health in diabetics.

From these studies we cannot determine whether the onset of diabetes precedes CAC depletion or whether the lack of CACs promotes the diabetic phenotype. These questions are more easily addressed in animal studies, and in this regard, a genetic deletion of the insulin receptor in mice has also been associated with decreased levels of angiogenic progenitor cells and impaired vascular repair [30]. Additional causality and mechanistic studies are needed to more clearly define the relationship between CACs and diabetes in humans. Nevertheless, indications from prior human and animal studies are consistent with the idea that attenuated vascular repair due to low levels of pro-angiogenic cells, or dysfunctional pro-angiogenic cells promotes endothelial dysfunction and pathological outcomes such as retinopathy [29], neuropathy [11, 35], and nephropathy [10, 36].

The decrease of CACs in diabetes has been attributed in part to the defective mobilization of these cells from the bone marrow. Several mechanisms have been proposed to account for this including aberrant PKA activation [37], deficits of upstream mobilizing agonists and signaling molecules such as SDF-1α, VEGF and nitric oxide [25, 33, 38], and impaired β-adrenergic responsiveness and neuropathy [39, 40]. Furthermore, there is evidence that the release of these cells is regulated in a circadian manner [41–43] and it has been recently reported that the diurnal release of CD34^+^ cells with high levels of ASM is disrupted with rhythmicity loss in diabetics [29]. In addition, our results, at least with the CAC-3 subtype, suggest that decreased expression of CXCR4 and VEGFR2, receptors for the potent mobilizing agents SDF-1α and VEGF, may also contribute to the impaired mobilization of these cells. Thus, even in the presence of high levels of these mobilizing agonists induced by injury or ischemia, CAC-3 cells in diabetics may have limited responsiveness to these mobilizing cues. We also found that a larger percentage of diabetics have high levels of pro-angiogenic cytokines and signaling molecules (Table 5), released perhaps as a result of persistent diabetes-induced injury. This observation further supports the idea that impaired angiogenesis in diabetics may be a consequence of response defects in CACs. Thus hyperglycemia may impact multiple pathways contributing to CAC availability.

One of the unexpected outcomes in our analysis was the association of cell counts with race. In the unadjusted model, a higher percentage of African Americans were in the high cell count group compared to other ethnicities. However, when stratified by race, 90% of African Americans in the low cell group had diabetes while only 50% of the Caucasians in the low cell group had diabetes. Therefore, the predictive power of CAC-3 cells for the onset of diabetes may be limited somewhat by race. While the basis for these racial differences is unknown, African Americans in the low cell group of our cohort may be subject to additional health, environmental, or socioeconomic factors which contribute to the onset or progression of diabetes. Interestingly, other race-related quantitative differences in vascular reparative cells have been described. Specifically, Caucasians had fewer CD14/CD16/KDR cells than did African-Caribbeans but more CD14/CD16/CD34 cells [44]. Furthermore, South Asians were found to have fewer CD34/CD133/KDR cells [45] and CD34/KDR [46] cells than did Caucasians.

In summary, we have found that low levels of several CAC subtypes were predictive of diabetes and that one in particular, characterized as CD34^+^/AC133^+^/CD31^+^/CD45^dim^, was indicative of both diabetes and endothelial dysfunction. Quantitative assessments of this biomarker may allow prognostic indications of disease onset and severity and might be effectively used to determine treatment options to offset adverse outcomes in this growing world-wide epidemic.

## Supporting information

S1 Table CAC description. (DOCX)

S2 Table Race, CAC-3 and diabetes. (DOCX)
