# Supplementary material for: Circulating angiogenic stem cells in type 2 diabetes are associated with glycemic control and endothelial dysfunction
Source: PLoS One. 2018 Oct 15;13(10):e0205851. doi: 10.1371/journal.pone.0205851 (PMC6188890; doi:10.1371/journal.pone.0205851)
Supplement: S1 Table — (DOCX) [file pone.0205851.s001.docx]

**S1 Table. CAC description**

| CAC | Phenotype |
| --- | --- |
| CAC-1 | CD31^+^/CD34^+^/CD45^dim^ |
| CAC-2 | CD45^+^/CD34^+^/CD31^+^ |
| CAC-3 | CD31^+^/CD34^+^/AC133^+^/CD45^dim^ |
| CAC-4 | AC133^+^/CD34^+^/CD31^+^/CD45^+^ |
| CAC-5 | AC133^+^/CD31^+^ |
| CAC-6 | CD31^+^/CD34^+^ |
| CAC-7 | CD31^+^/CD34^+^/AC133^-^/CD45^dim^ |
| CAC-8 | AC133^-^/CD34^+^/CD31^+^/CD45^+^ |
| CAC-9 | CD34^+^ |
| CAC-10 | CD31^+^ |
| CAC-11 | AC133^+^ |
| CAC-12 | CD45^+^ |
| CAC-13 | CD34^+^/AC133^+^ |
| CAC-14 | AC133^+^/CD34^+^/CD45^+^ |
| CAC-15 | AC133^+^/CD34^+^/CD45^dim^ |
| CAC-16 | KDR^+^ |
| CAC-17 | KDR^+^/CD34^+^ |
| CAC-18 | KDR^+^/CD34^+^/AC133^+^ |
