# Supplementary material for: Circulating angiogenic stem cells in type 2 diabetes are associated with glycemic control and endothelial dysfunction
Source: PLoS One. 2018 Oct 15;13(10):e0205851. doi: 10.1371/journal.pone.0205851 (PMC6188890; doi:10.1371/journal.pone.0205851)
Supplement: S2 Table — (DOCX) [file pone.0205851.s002.docx]

**S2 Table. Race, CAC-3 and diabetes**

| CAC-3 level | Non-diabetic  n (% of total) | Diabetic  n (% of total) | P value |
| --- | --- | --- | --- |
| African Americans | | | |
| Low count | 1 (10) | 9 (90) | 0.007 |
| High count | 13 (62) | 8 (38) |  |
| Caucasians |  |  |  |
| Low count | 17 (50) | 17 (50) | 0.259 |
| High count | 18 (64) | 10 (36) |  |
